# Development of a sensitive and specific xMAP assay for detection of antibodies against infectious laryngotracheitis and bronchitis viruses

**DOI:** 10.1186/s12985-018-1048-x

**Published:** 2018-09-21

**Authors:** Huanan Wang, Feng Cong, Jianchi Guan, Li Xiao, Yujun Zhu, Yuexiao Lian, Ren Huang, Meili Chen, Pengju Guo

**Affiliations:** 1 Department of Veterinary Medicine, College of Animal Sciences, Zhejiang University, Hangzhou, 310058 China; 2 Guangdong Laboratory Animals Monitoring Institute and Guangdong Provincial Key Laboratory of Laboratory Animals, Guangzhou, 510640 China

**Keywords:** Luminex xMAP technology, Infectious laryngotracheitis virus, Infectious bronchitis virus, antibody detection, ELISA

## Abstract

**Background:**

A serological method to simultaneously detect antibodies against infectious laryngotracheitis virus (ILTV) and infectious bronchitis virus (IBV) is imperative for the differential diagnosis and evaluation of antibodies titers after vaccination.

**Method:**

The microspheres coated with purified recombinant glycoprotein D (gD) of ILTV or nucleocapsid (N) protein of IBV were incubated with serum samples. The simultaneous quantification of ILTV and IBV antibodies were achieved through the interrogation of microspheres by Luminex 200 detection system. .

**Results:**

This xMAP detection demonstrated no nonspecific reactions with avian influenza virus (AIV), avian leukosis virus (ALV), newcastle disease virus (NDV), and Marek’s disease virus (MDV). The results also demonstrated that the xMAP assay was four times more sensitive than the enzyme-linked immunosorbent assay (ELISA) for ILTV detection and two times more sensitive for IBV detection. A total of 90 chicken serum samples from a chicken farm were tested by xMAP and ELISA assays. The results showed that the coincidence rates were 84.44 and 100% for ILTV and IBV detection, respectively.

**Conclusion:**

This study exhibited an opportunity for the differential diagnosis through simultaneous detection of multiplex antibodies in serum and can be used for the multiplex antibodies evaluation after vaccination.

## Background

Infectious laryngotracheitis (ILT) and infectious bronchitis (IB) are common respiratory diseases in poultry and are currently present at epidemic levels around the world, including in China, the United States, and India. Because ILT and IB have high incidence and infectivity in chickens at different ages (in days), they have caused huge economic losses to the poultry industry. Both diseases have similar clinical symptoms and pathological changes, leading to significant difficulties in clinical differential diagnosis, especially in cases of mixed infection [1, 2]. In addition, the use of vaccines is the main approach to control of the economically important poultry viral respiratory diseases, such as infectious laryngotracheitis and infectious bronchitis [3]. Therefore, it is important to establish a method to simultaneously detect ILTV and IBV antibodies for the differential diagnosis and immune response evaluation after vaccination.

Infectious laryngotracheitis virus (ILTV), an alphaherpes virus, possesses at least 10 envelope glycoprotein, two main proteins of which are the glycoprotein B (gB) and gD, respectively, which are highly conserved herpesvirus structural glycoproteins [4]. The gD of herpes virus has an important role by binding to the host receptors [1, 5]. The gD protein has been demonstrated to be a candidate antigen for recombinant vaccines [6, 7]. The infectious bronchitis virus (IBV) genome encodes four major structural proteins: the spike glycoprotein (S), the membrane glycoprotein (M), the nucleocapsid (N) protein, and the envelope or small membrane protein (E) [8]. The N protein is thought to be an appropriate diagnostic reagent for antibody detection [9]. In this study, gD and N proteins were selected as antigen molecules for the diagnosis of ILT and IB.

At present, the methods used for the diagnosis and effect evaluation of vaccine immunity of avian respiratory diseases primarily include virology detection, serological detection, and molecular biological detection. Traditional methods, such as virus isolation, animal inoculation experiments, ELISA, hemagglutination (HA), and hemagglutination inhibition (HI) assays are characterized by complex procedures and long periods of time required for diagnosis. Despite its unique, sensitive, simple features and rapidity, polymerase chain reaction (PCR) is unable to meet the requirements for high-flux quarantine and is not favorable for the urgent screening of bulk samples. Therefore, it is imperative to establish a new technology for the effect evaluation of vaccine immunity and the rapid differentiation or correct identification of important avian respiratory diseases.

A new high-flux detection technology, flexible xMAP (x = Unknown, MAP = Multi-Analyte Profiling) assay, uses variously colored polystyrene beads that is carboxylated to allow covalent coupling of protein. Conjugated beads can be used to capture specific antibodies in serum, and a fluorescent secondary antibody is incubated to bind to the captured serum antibodies. A red laser is used to determine the color of the bead and a green laser to detect fluorescence intensity of bound secondary antibodies through the Luminex 200 detection system [10]. This method is applicable for the simultaneous and rapid detection of multiplex pathogen^,^s antibodies, with simple operation and high accuracy that are superior to conventional methods. In this study, a method employing Luminex xMAP technology to simultaneously detect ILTV and IBV antibodies in serum was established, optimized and used for the differential diagnosis of IBV and ILTV. This assay can also be used to simultaneous monitoring of IBV and ILTV antibody levels for the evaluation of immunity after vaccination.

## Results

### Establishment of singleplex xMAP detection for ILTV and IBV

A singleplex xMAP for ILTV was established by testing six concentrations of the ILTV gD envelope protein (2.5 μg, 5 μg, 10 μg, 20 μg, 40 μg, and 80 μg per 5 × 10^5^ magbeads), seen in Table 1. The P/N value is the ratio of MFI (Median Fluorescence Intensity) value between the positive sample and negative sample. The optimal conjugation protein concentration was 2.5 μg per 5 × 10^5^ magbeads for singleplex xMAP detection of ILTV. The mean P/N value under the condition was 10.18.

**Table 1:** P/N values for ILTV antibody xMAP detection

Serum dilution	Coating antigen dilutions (μg/5 × 10^5^ beads)						Average
	2.5	5	10	20	40	80	
1:25	16.84	8.41	11.18	10.15	6.87	3.22	9.44
1:50	10.47	6.26	8.54	7.39	4.43	2.70	6.63
1:100	8.30	5.07	5.84	6.11	4.07	2.69	5.35
1:200	5.14	3.88	4.83	4.55	2.97	2.21	3.93
Average	10.18	5.90	7.60	7.05	4.59	2.70	

A singleplex xMAP for IBV was established by testing six concentrations of the IBV N envelope protein (3 μg, 6 μg, 12 μg, 24 μg, 48 μg, and 96 μg per 5 × 10^5^ magbeads), seen in Table 2. The P/N value is the ratio of MFI value between the positive sample and negative sample. The optimal conjugation protein concentration was 12 μg per 5 × 10^5^ magbeads for singleplex xMAP detection of IBV. The mean P/N value under the condition was 17.16.

**Table 2:** P/N values for IBV antibody xMAP detection

Serum dilution	Coating antigen dilutions (μg/5 × 10^5^ beads)						Average
	3	6	12	24	48	96	
1:50	13.16	14.67	17.53	14.75	16.30	13.30	14.95
1:100	12.23	18.15	16.44	16.07	18.83	17.66	16.57
1:200	13.41	17.07	17.73	14.63	15.16	17.52	15.92
1:400	11.59	14.72	16.93	11.88	12.30	14.90	13.72
Average	12.60	16.15	17.16	14.33	15.65	15.85	

### Establishment of duplex xMAP for simultaneous detection of ILTV and IBV

**Fig. 1:**
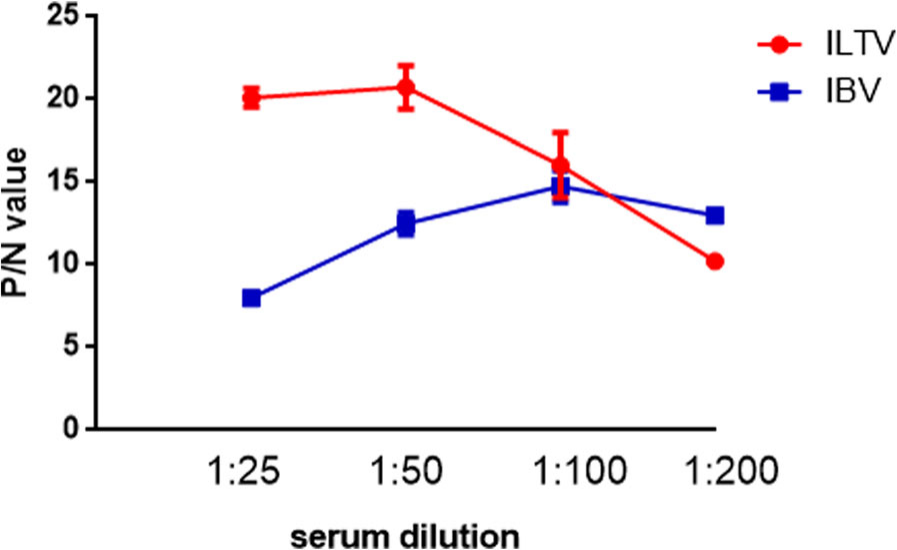
Determination of optimal serum dilution ratio for duplex xMAP assay for ILTV and IBV antibodies. ILTV- and IBV-positive and negative sera were diluted at the range from 1:25 to 1:200. MFI Values were obtained after the interrogation of microspheres coated with proteins of ILTV or IBV using Luminex 200 detection system. The P/N values were used to determine the optimal dilution ratio at 1:100 for duplex detection of ILTV and IBV antibodies

Based on the results in Tables 1 and 2, the optimum serum dilution ratio for ILTV and IBV were 1:25 and 1:100, respectively. When singleplex xMAP assay was performed. To determine the optimal serum dilition ration for simultaneous detection of ILTV and IBV, a serial serum dilutions from the range of 1:25 to 1:200 were prepared and were incubated with ILTV and IBV antigen conjugated microspheres. From the P/N values showed in Fig. 1, it is obvious that the optimal serum dilution ratio for duplex xMAP assay was 1:100.

### Determination of threshold

The threshold is considered as the sum of the average plus three times of standard deviation, which is also named cut-off value. To determine the threshold of xMAP assay for ILTV and IBV, 32 special pathogen free chicken serum samples at 1:100 dilution were used to obtain the average MFI value and standard deviation. From the Table 3, it showed that the threshold of ILTV and IBV detection were 154.95 and 266.75, respectively.

**Table 3:** Threshold of ILTV and IBV antibodies detection

	ILTV MFI value				IBV MFI value			
SPF chicken negative sera	113	101.5	105	93	207.5	198.5	196.5	184
	105	97	110	134	192.5	197	222.5	258
	68	68	70	74.5	127	132	134.5	149.5
	58	67	68	72	116.5	134	135	141.5
	106	115.5	75	90	183	152.5	148	134
	126	98	104.5	111	140	158.5	164	116
	89.5	86	77	86	132.5	150	153	144
	48.5	59.5	43	70	119	95	132	87
Average	87.17				154.22			
Standard deviation	22.59				37.51			
Threshold	154.95				266.75			

### Specificity of xMAP assay for ILTV and IBV

**Fig. 2:**
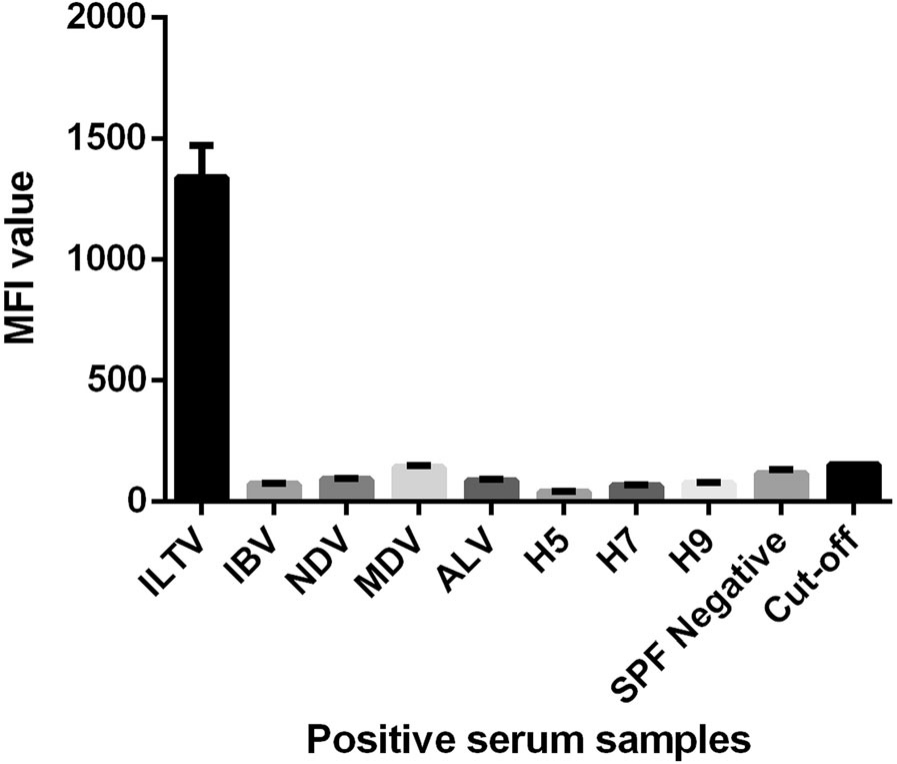
Specificity for duplex xMAP assays for ILTV antibody detection. Positive sera for ILTV, IBV, ALV, NDV, MDV, AIV(H5, H7 and H9) were used to determine the specificity of duplex xMAP assay. The specific detection results showed that there were no cross-reactions with positive sera for other avian diseases

**Fig. 3:**
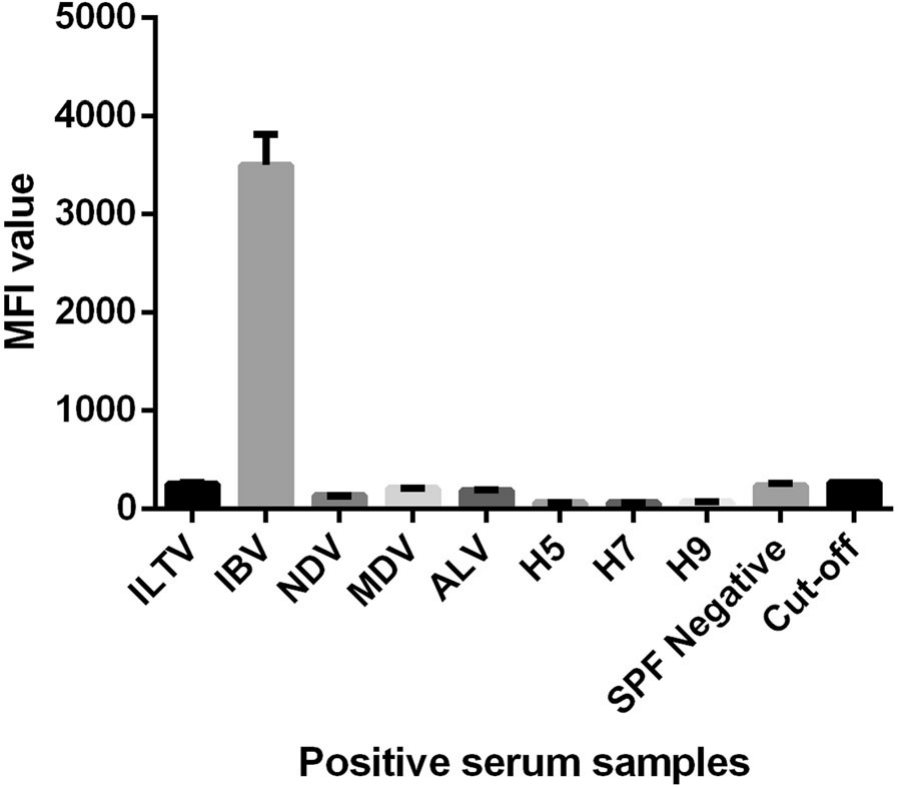
Specificity for duplex xMAP assays for IBV antibody detection. Positive sera for ILTV, IBV, ALV, NDV, MDV, AIV were used to determine the specificity of duplex xMAP assay. The specific detection results showed that there were no cross-reactions with positive sera for other avian diseases

The results in Figs. 2 and 3 demonstrated that xMAP duplex assay for ILTV and IBV has a high specificity since there were no cross-reactions with serum positive for other avian diseases, such as avian influenza virus (AIV), avian leukosis virus (ALV), newcastle disease virus (NDV), and Marek’s disease virus (MDV).

### Repeatability of xMAP assay for ILTV and IBV

Intra-assay repeatability experiments showed that the coefficient of variation (CV) was 5.09% for ILTV and 8.05% for IBV. Inter-assay repeatability experiments showed that the CV was 8.11% for ILTV and 9.48% for IBV. All CVs were less than 10%, indicating that the method has high repeatability. The results were shown in Tables 4 and 5.

**Table 4:** Repeatability experiment of ILTV antibody detection

ILTV detection	Samples	MFI	Average	Standard deviation	Intra Cv
Repeat Experiment 1	repeat 1	1145.5	1208.33	61.5433	5.09%
	repeat 2	1268.5			
	repeat 3	1211			
	Negative	111.33	/	/	/
Repeat Experiment 2	repeat 1	1127.5	1143.00	55.8860	4.89%
	repeat 2	1205			
	repeat 3	1096.5			
	Negative	84.33	/	/	/
Repeat Experiment 3	repeat 1	1046	1028.00	16.3707	1.59%
	repeat 2	1024			
	repeat 3	1014			
	Negative	75.33	/	/	/
Inter MFI Average		1126.44			
Standard deviation		91.30			
Inter Cv		8.11%

**Table 5:** Repeatability experiment of IBV antibody detection

IBV	Samples	MFI	Average	Standard deviation	Intra Cv
Repeat Experiment 1	repeat 1	2096.5	2177.67	75.2103	3.45%
	repeat 2	2245			
	repeat 3	2191.5			
	Negative	177	/	/	/
Repeat Experiment 2	repeat 1	1770	1884.50	151.6583	8.05%
	repeat 2	2056.5			
	repeat 3	1827			
	Negative	141.33	/	/	/
Repeat Experiment 3	repeat 1	1842	1832.00	106.3532	5.81%
	repeat 2	1721			
	repeat 3	1933			
	Negative	127	/	/	/
Inter MFI Average		1964.72			
Standard deviation		186.27			
Inter Cv		9.48%			

### Comparison of xMAP assay with ELISA using clinical samples

The sensitivities of xMAP and ELISA were compared using 1:50 to 1:3200 ILTV-positive sera and 1:100 to 1:6400 IBV-positive sera. The results showed that xMAP detected ILTV-positive sera at 1:1600 and IBV-positive sera at 1:3200, while ELISA detected ILTV-positive sera at 1:400 and IBV-positive sera at 1:1600 (see Table 6).

**Table 6:** Comparison of xMAP and ELISA sensitivity

ILTV detection					IBV detection				
Dilution	ELISA S/P		xMAP MFI		Dilution	ELISA S/P		xMAP MFI	
1:50	2.9178	+	2205	+	1:100	1.5430	+	2732.5	+
1:100	2.0395	+	1725.5	+	1:200	1.0445	+	2103	+
1:200	1.3866	+	1235	+	1:400	0.6319	+	1960	+
1:400	0.8119	+	806.5	+	1:800	0.3961	+	1161	+
1:800	0.4607	–	486.5	+	1:1600	0.2301	+	779.5	+
1:1600	0.2536	–	277.5	+	1:3200	0.1372	–	428.5	+
1:3200	0.1262	–	145	–	1:6400	0.0828	–	226	–
Cut-off	0.5000		154.95		Cut-off	0.2000		266.75	

To compare duplex xMAP assay with ELISA for ILTV/IBV detection, 90 chicken serum samples from a chicken farm in Huizhou, China were used and the results are shown in Table 7. For ILTV, xMAP detected 76 positive cases whereas ELISA detected 69 positive cases; among them, 67 samples were detected positive by xMAP assay and ELISA. All 90 samples were detected as positive for IBV by both methods.

**Table 7:** Comparison of xMAP and ELISA for ILTV and IBV antibody detection

	Detection method	Positive sample	Negative samples	Total coincidence
ILTV	xMAP	76 (67^a^)	14 (9^a^)	84.44%
	ELISA	69	21	
IBV	xMAP	90 (90^a^)	0 (0^a^)	100.00%
	ELISA	90	0	

^a^Identical number of detection results for xMAP and ELISA

## Discussion

IB, an acute infectious disease characterized by acute watery diarrhea, kidney necrosis, and high mortality, is caused by IBV. Thus far, IB has resulted in economic losses to the poultry industry in various countries [8, 11]. IBV infects chickens at different ages. Chicks infected with IBV are characterized by noisy breathing, panting, sneezing, nasal fluid, and other symptoms; furthermore, laying hens exhibit significantly reduced egg production, broilers exhibits low growth and bad meat quality, and breeding hens exhibit significantly reduced breeding efficiency [12]. ILT is an acute, contact respiratory infectious disease in chickens. Chickens infected with ILT are characterized by nasal fluid, conjunctivitis, panting, coughing, and the production of bloody mucus, as well as swelling, erosion, necrosis, and extensive bleeding in the throat after analysis; laying hens exhibit reduced egg production or even no production, as well as poor egg quality [13]. The disease spreads rapidly and has a wide distribution. The high mortality rate (> 70%) results in huge economic losses to the poultry industry [4]. Given the similar clinical symptoms between ILT and IB, the development of diagnoses for both diseases is significant.

This is the first case report of differential diagnosis of ILTV and IBV with xMAP assay. Luminex xMAP assay is an emerging technology that uses small carboxylated polystyrene microspheres that are internally dyed with both a red and an infrared fluorophore [14]. By changing the ratio of the two fluorophores, it is possible to distinguish up to 100 different color-coded microsphere sets, and each microsphere set may be coupled with a different biological probe. The microspheres are detected and characterized by a dedicated flow cytometer [15]. The Luminex xMAP technology is useful for many different applications. One review describes the use of this technology for the multiplex detection of viral, bacterial, parasitic, and fungal agents using the microsphere-based multiplex nucleic acid assay (MBMNA) and the microsphere-based multiplex immunoassay (MBMI) [16]. MBMIs are typically biochemical tests that allow the detection or measurement of the concentration of a protein in a solution via an antibody or immunoglobulin [17]. MBMIs are often used in the diagnosis of various pathogens, such as human papillomavirus and human cytomegalovirus, to detect antibodies in serum samples [18–20]. The advantages of this technology are its simple operation, high accuracy, and reduction in the number required experiments and samples because a single test simultaneously identifies a variety of pathogens.

This study describes the establishment and validation of single xMAP and duplex xMAP assays. First, to achieve effective and rapid clinical diagnosis of ILT and IBV, single xMAPs were established for the separate diagnosis of these two diseases after screening to identify the best concentrations of microsphere antigens for ILTV gD and IBV N (2.5 μg/5 × 10^5^ magbeads and and 12 μg/5 × 10^5^ magbeads, respectively), with an optimal serum dilution of 1:100. However, it is not ideal to diagnose only one type of pathogen antibody because more than one disease often affects the respiratory tract in poultry. Given the similar clinical symptoms of ILT and IBV, the differential diagnosis of these two pathogens with two single xMAP assays using one sample wastes time and energy. To achieve the rapid differential diagnosis of clinical samples, it is necessary to establish a duplex xMAP. In addition to ILTV and IBV, AIV and NDV are also common respiratory viruses for poultry. These four diseases have high incidences, and it is difficult to make a differential diagnosis based on clinical symptoms, which are all similar. Figures 2 and 3 showed that our xMAP methods was able to simultaneously detect the antibodies to ILTV or IBV in the sera and had the high specificity. A duplex xMAP can quickly identify ILTV and IBV infection, but it does not indicate possible infection by AIV and NDV. Therefore, based on this study, we propose that quadruplex xMAP should be established in the future for the detection of ILTV, IBV, AIV and NDV as a fast and effective differentiation tool for these four viruses.

Since ELISA was established to detect ILTV antibodies in 1982 [21], it has demonstrated strong advantages, as well as higher sensitivity than virus neutralization tests (VNT), making it widely applicable. ELISA antibody detection of IBV is also feasible at earlier time points [22, 23] and is currently broadly applied in the poultry industry. Single-analyte ELISA does not support the detection of multiple specific antibody responses simultaneously for a single serum sample [24] and possesses other disadvantages, such as the requirement for a relatively large amount of sample, negligible nonspecific binding, and increased background. However, with the development of the poultry industry, requirements dictate that many samples must be detected simultaneously; thus, a new detection method for diagnosis with ILTV and IBV is significant. Luminex xMAP assay represents an alternative for commonly employed indirect tests such as ELISA. The conversion of an ELISA to the xMAP format is uncomplicated, efficient and cost-saving, producing an assay with superior dynamic range and sensitivity [25]. MBMI represents increased sensitivity and the potential to quantify antibodies, antigens, and other substances (e.g., hormones, cytokines, and tumor markers), unlike conventional ELISA tests [26]. Luminex xMAP has also been compared with a western blot assay for antibody detection [27]. The results obtained from both methods showed that the sensitivity of xMAP was 82.7%, while the western blot assay sensitivity was 74.7%. Therefore, xMAP may be more efficient and precise than western blots for the diagnosis of diseases. Although ELISA provides relatively accurate results and has been used for many years, this study demonstrates that xMAP is much more sensitive than ELISA for the diagnosis of ILT, as indicated by the results presented in Table 6. In addition, xMAP may be used for the rapid diagnosis of many samples, saving time and effort. The results presented in Table 7, demonstrate that xMAP is highly accurate, with similar detection results to those obtained by ELISA in positive clinical samples; thus, xMAP is able to be widely applicable.

## Conclusions

This study describes how to determine the optimal conditions for establishing duplex xMAP assay based on the singleplex xMAP result, thus providing a reference for establishing multiplex xMAP assay for simultaneous detection of animal pathogen^,^s.antibodies.

## Methods

### Antigens and sera

The antigens used for ILTV were a recombinant gD [28], expressed, purified, preserved in our laboratory. For the detection of antibodies against IBV, the recombinant N protein was expressed as GST fusion protein in *Escherichia coli* BL21 (DE3) cells [29]. The negative sera of SPF chickens and the positive virus serum (ILTV, ALV, NDV, MDV, H5, H7 and H9) were purchased form Harbin Weike Biological Technology Development Company, China. The positive sera (IBV and AIV) were obtained from China Veterinary Culture Collection Center. Ninety of clinical chicken serum samples were collected from a chicken farm in Huizhou, China.

### Coupling of proteins to fluorescent microspheres

Prior to coupling, the recombinant proteins was desalted by gel filtration using MicroBio-Spin 6 columns (Bio-Rad, California, USA) based on the manufacture’s protocol to exchange the buffer from sodium azide or imidazole to PBS. All the antigens were quantified using Pierce BCA protein Quantification Kit (Thermo Scientific, USA). Coupling of proteins to fluorescent microspheres was performed as described by Karanikola et al. [30]. MAP MC10007 beads (Luminex, USA) were coupled by gD protein of ILTV and MAP MC10015 beads (Luminex, USA) were coupled by N protein of IBV. A certain number of beads was transferred to one coupling reaction tube, followed by 100 μL bead wash buffer and suspended in 80 μL bead activation buffer. Then 10 μL of 50 mg/ml EDAC (Sigma-Aldrich) and 10 μL of 50 mg/ml S-NHS (Sigma-Aldrich, Germany) were added in the reaction buffer. The reaction tube was gently vortexed for 20 min at room temperature (RT). PBS (pH 7.4, 150 μL) was added twice and the recombinant protein was added. Incubation was carried out at RT for 2 h. The coupled beads were centrifuged and washed. Ultimately, the beads were stored at 4 °C in 150 μL storage buffer in the darkness.

### Methods for bead-based xMAP technology

The xMAP assays were carried out in 96-well polystyrene microplates using Luminex 200 detection system (Luminex, USA). Relevant positive and negative sera have been prepared for the establishment of the singleplex xMAP assay to detect ILTV antibody or IBV antibody in the serum. ILTV gD protein at the following concentrations (2.5 μg, 5 μg, 10 μg, 20 μg, 40 μg, and 80 μg) and IBV N protein at the following concentrations (3 μg, 6 μg, 12 μg, 24 μg, 48 μg, and 96 μg) were coupled with 5 × 10^5^ magbeads to determine the optimum antigen concentration for singleplex xMAP assay. A serious dilution of sera at the range from 1:25 to 1:200 have been made to determine the optimal sample dilution. Cross-reactivity or specificity was evaluated by making the assay with sera from chicken infected with AIV, ALV, NDV, and MDV. Then, a duplex xMAP assay was established based on the result of two separated singleplex xMAP assay. In briefly, a 50 μL aliquot of the simplex beads (50 beads/μL) or the duplex beads (100 beads/μL, 50 beads/μL each kind) was transferred into the wells, added in 50 μL of diluted sera. Incubation was conducted on a plate shaker (800 rpm) at RT for 60 min. The centrifugation was conducted in the magnetic separator for 60 s. The supernatant was removed from each well. Beads were washed 5 times in 100 μL PBS. Each well was added with 100 μL of a biotinylated secondary antibody (goat anti-chicken IgY, Beijing Solarbio Science & Technology, China) at 2 μg/mL. The plate was shaken (800 rpm) for 30 min at RT and washed as described above. Finally, 2 μg/mL of streptavidin-phycoerythrin (SAPE, Life Technologies GmbH) was added to each well at 100 μL/well. The plate was shaken and the supernatant was removed. Finally, 100 μL of assay buffer was added to each well. The plate was shaken for approximately 15 s and analyzed according to the manufacturer’s protocol. All samples were conducted in triplicates in each assay.

### ELISA used for comparison

Commercial ILTV and IBV ELISA kit (Biochek, Netherlands) were used to detect presence of ILTV or IBV antibodies in poultry sera. The assay was performed according to the manufacturer’s protocols.

## Abbreviations

AIV: Avian influenza virus
ALV: Svian leukosis virus
IB: Infectious bronchitis
ILT: Infectious laryngotracheitis
MDV: Marek’s disease virus
NDV: Newcastle disease virus
